# Edaphic and light conditions of sympatric plant morphotypes in western Amazonia

**DOI:** 10.3897/BDJ.2.e1078

**Published:** 2014-05-10

**Authors:** Julissa Roncal

**Affiliations:** † Memorial University of Newfoundland, St. John's, Canada

**Keywords:** Canopy openness, floodplain, *Geonoma macrostachys*, habitat differentiation, leaf area index, Peru, slope, soil texture, soil nutrients, terra firme, transmitted light, tropical rainforest

## Abstract

Here I present a dataset of edaphic and light conditions associated with the occurrence of sympatric morphotypes of *Geonoma macrostachys* (Arecaceae/Palmae), a candidate case study from Amazonia hypothesized to have evolved under ecological speciation. Transects were established in three lowland rainforests in Peru, and the abundance of each local morphotype of this species was recorded in a total area of 4.95 hectares. Composite soil samples and hemispherical photographs were taken along the transects were the species occurred to obtain information on soil nutrients, soil texture, and indirect measurements of light availability. The raw and summary tables disclose the characteristics of each study site and habitats within them, which could be useful to soil scientists, ecologists, and conservationists engaged in similar research activities or meta-analyses in Amazonia.

## Introduction

It is well known that soil chemistry, texture, and topography can determine the plant community composition and species richness at different spatial scales (e.g. Gentry 1981, Eiserhardt et al. 2011). For example, the turnover of community species composition along a soil fertility gradient has been documented at local and regional scales (e.g. Poulsen et al. 2006, Andersen et al. 2010, Guèze et al. 2013). Plant species grow preferentially under different soil nutrient concentrations and textures (e.g. John et al. 2007, Baribault et al. 2012). Flooding versus good drainage also affects plant distribution (e.g. Silvertown et al. 1999, Duque et al. 2002). Soil texture is related to drainage, and it characterizes the bulk density, surface area, and air space in between soil particles, affecting the water-holding capacity and hydraulic conductivity of soils (Rawls et al. 1982, Sollins 1998, Palm et al. 2007). Topography also influences species distributions through its interaction with other environmental factors such as soil nutrients, hydrology, wind exposure, temperature and even biotic factors (Trichon 1997, Pausas and Austin 2001, Klinger and Rejmánek 2010). Its effect on plant performance is thus indirect, difficult to interpret and often site specific (Vormisto et al. 2004). Although less studied, the distributions of many plant species show strong associations with light availability (e.g. Terborgh and Mathews 1999). The vertical distribution of foliage in a forest allows light to penetrate the understory through vertical and lateral gaps of different sizes, creating a vertical and horizontal light heterogeneity in the forest understory (Oberbauer et al. 1989, Montgomery 2004) that could allow resource partitioning among species. These plant responses to abiotic conditions suggest an important role for habitat heterogeneity not only as a mechanism that facilitates the coexistance of high species diversity, but also as a speciation driver (e.g. Gentry 1989, Haffer 1997, Nosil 2012). Documentation of habitat heterogeneity should thus be an important component in biodiversity studies.

Nosil (2012) defined ecological speciation as the process by which barriers to gene flow evolve between populations as a result of ecologically based divergent selection between environments. The interaction of individuals with their environment is thus a key agent of selection under this mode of speciation, making the documentation of habitat preferences between populations an important observation (yet not the only one) to empirically distinguish ecological speciation. The palm species complex, *Geonoma macrostachys* Mart. (Arecaceae), is a potential case study of ecological speciation in western Amazonia. Local morphotypes of this lowland forest palm differ in leaf shape, show a strong habitat differentiation, are reproductively isolated by differences in pollinator guild and flower phenology while genetic data suggest an independent evolution of the morphotypes in each forest site (Listabarth 1993, Roncal 2005, Roncal 2006, Roncal et al. 2007).

Here, I present a dataset of edaphic and light properties that were used to determine the presence and degree of habitat differentiation between local morphotypes of *Geonoma macrostachys* in three lowland moist forests in Peru (Roncal 2005, Roncal 2006). These publications did not make the raw data available. Following Svenning (1999), I define habitat as the environmental conditions occurring at the scale of a floodplain or terra firme (i.e. more than one km^2^). I refer to microhabitat as those characteristics within major habitat types that change at scales less than 10^3^ m (Svenning 1999). This information could complement similar environmental studies spanning the distribution range of this palm species in order to test more rigorously the ecological speciation hypothesis in Amazonian plants. Finally, the environmental data available here could be useful to soil scientists, ecologists, and conservationists who seek detailed environmental information at the habitat and microhabitat scales for this part of the Amazon basin.

## Project description

### Title

Habitat differentiation of sympatric *Geonoma macrostachys* (Arecaceae) morphotypes in Peruvian lowland forests

### Personnel

Julissa Roncal

### Study area description

**Figure 1.**
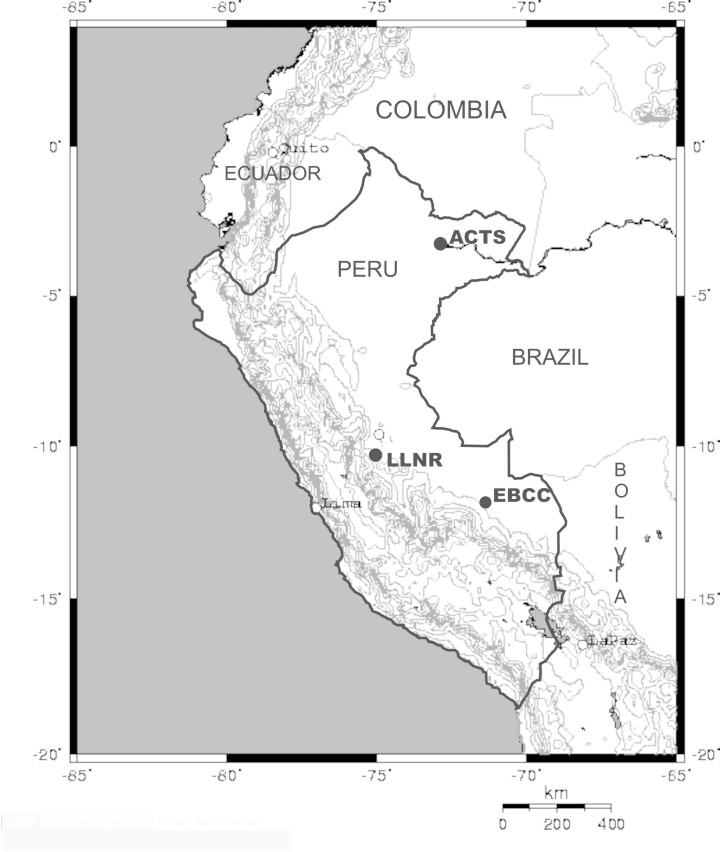
Map of the three study sites in Peru where soil and light conditions were measured. Locality acronyms are the same as in Table 1.

Fieldwork was carried out at three sites. The Amazon Conservatory of Tropical Studies (ACTS) is situated adjacent to the Sucusari, a small tributary to the Napo River in northeast Peru. ACTS is located within the Explornapo Reserve, a 1,725 ha of mostly primary forest, property of Explorama Tours (Vasquez 1997). Soils in the reserve belong to the Pebas formation, which dates back to the Middle Miocene (Hoorn 1994), and gave rise to clay and silty clay soils with a higher than average nutrient content (Vasquez 1997, Vormisto et al. 2004). Most of the reserve is covered by terra firme forest but the area adjacent to the Sucusari was classified as Igapo or floodplain. For a detailed description of the floristic composition of the area see (Vasquez 1997). The Loma Linda Native Reserve (LLNR) is a 332.16 ha protected area located adjacent to the Palcazu River in central Peru. No information on the geology or soil type of the reserve has been published. Two main habitat types were visually recognized in the field: a topographically irregular red-soil habitat, and a flat white-soil habitat. Finally, the 1,000 ha study area of Cocha Cashu biological station (EBCC) is located within the lowlands of the 1,532,000 ha of Manu National Park in southeastern Peru (Terborgh 1990). Soils at EBCC within the 6 km-wide meander belt of the Manu River (floodplain forest) are composed of young alluvial silt and clay carried from the Andes. Soils in the uplands (terra firme) of EBCC, dissected by numerous streams, are sandy (Terborgh 1990). Foster (1990) described the floristic composition of the Manu river floodplain forests. Table 1, Fig. 1.

### Funding

The Marina Riley Scholarship Program of Duke University, the International Palm Society, the South Florida Palm Society, the Karling graduate student award of the Botanical Society of America, the Tropical Biology Program of Florida International University.

## Sampling methods

### Sampling description

At each site, transects of 10 m wide and 290 m long were established on each main habitat described in the 'study site' section, and separated from one another by at least 200 m. Eleven, twelve, and fourteen transects were established at EBCC, LLNR, and ACTS, respectively. Transects were divided into plots of 10 m × 10 m and all *Geonoma macrostachys* adult individuals having the minimum reproductive height were recorded in every other plot to avoid spatial autocorrelation (Suppl. material 1). The position of transects are disclosed in Table 2. The total area sampled in this study was 4.95 hectares. A map of the trail system at ACTS can be found in Suppl. material 2, and a LANDSAT map, as well as the trail system at EBCC can be found in http://cochacashu.sandiegozooglobal.org/researchers/maps/.

The inclination of every other plot along each transect was measured with a clinometer (PM5/360PC, Suunto®, Finland) in the middle of the plot. Soil samples for laboratory analyses were taken from 78, 76, and 87 plots from ACTS, LLNR, and EBCC, respectively (241 soil samples in total). Plots were randomly chosen along transects so that at least 40 soil samples per morphotype at each site were collected with no more than nine soil samples per transect. Since at EBCC fewer than 40 plots were recorded to have the *Geonomaacaulis* morphotype, 17 additional soil samples were collected from haphazard *Geonomaacaulis* individuals in the forest. For the same reason, nine soil samples from haphazardly chosen *large morphotype* individuals were collected at LLNR. At each plot, the top 20 cm of soil profile (Ah horizon) was sampled at three points within a 0.5 m radius of the palm(s), using a 2.5 cm diameter × 30 cm high metallic cylinder, and mixed to obtain a composite soil sample. This procedure was also followed for plots where the two varieties were found, collecting only one composite sample.

**Figure 2.**
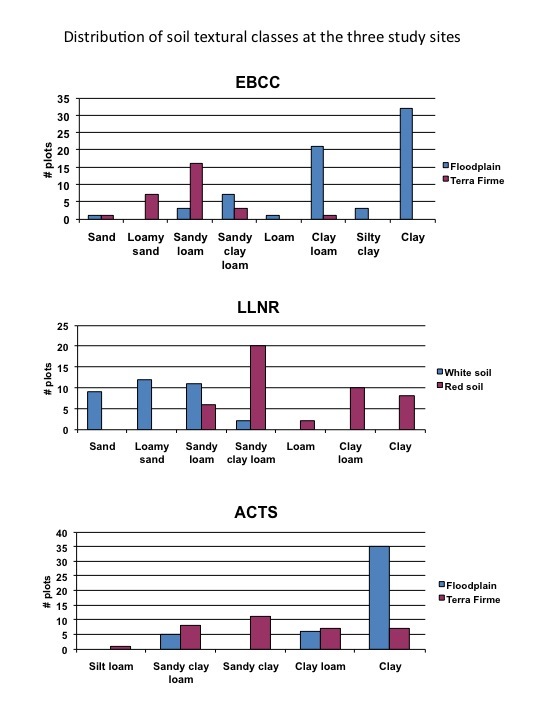
Distribution of soil textural classes at the three study sites following the USDA textural triangle system (Thein and Graveel 2002). Plots sampled from the main habitat types are distinguished on each case. Data used for these figures were obtained from Suppl. material 3.

Soil texture was quantified using a hydrometer, which calculates the proportional distribution of sand (particle size of 0.05 mm and larger), silt (0.002–0.05 mm) and clay (<0.002 mm) in the soil through the application of the Stoke’s law of mineral particle separation by size, based on the settling rate in suspension (Thein and Graveel 2002). Soils were further assigned to one of the 12 textural classes using the United States Department of Agriculture (USDA) textural triangle (Thein and Graveel 2002). Soil chemical analyses included pH using an electrode in a 1:1 solution of soil and water, and the following extractable cations: Ca, Mg, P, K, Zn, Mn, Cu, B, and Na, using the Mehlich 1 extractant and an Inductively Coupled Plasma (TJA 61E, Thermo Electron Corporation, Florida). These analyses were conducted at the Agricultural Service Laboratory of Clemson University. Suppl. material 3 presents the raw data. Table 3 is a summary table showing mean values and standard deviations for each main habitat within the study sites. Table 4 is another summary showing only the significantly different edaphic variables between morphotypes. Soil textural classes were also different between habitats at each site (Fig. 2). Clay and clay loam soils characterize the floodplain of EBCC and ACTS, while sandy soils characterize the terra firme at these sites. The white soil habitat at LLNR presents sand, loamy sand, and sandy loam, while the red soil habitat is mostly composed of sandy clay loam, clay loam and clay Fig. 2.

Hemispherical photographs were used to obtain an indirect measure of light availability for 40 palm individuals of each morphotype at each study site. Hemispherical photography is a technique used to estimate forest light conditions in the subcanopy and understory since light measurements obtained from this method correlated highly with direct measurements of photosynthetic photon flux density (Chazdon and Field 1987, Roxburgh and Kelly 1995, Machado and Reich 1999, Engelbrecht and Herz 2001). Individuals selected for this purpose were the same as those selected for soil analyses. I used a Nikon 8 mm fisheye lens (180° field of view) mounted on a Nikon COOLPIX 995 digital camera. Photographs were taken under uniformly overcast conditions (usually at dawn) to avoid reflection. The camera was oriented with a hand-held compass to ensure that a light emitting diode attached to the fisheye lens pointed the north, the camera was also leveled in a tripod before each photograph. Hemispheric photographs were analyzed with Gap Light Analyzer (GLA) software version 2.0 (Frazer et al. 1999, http://www.rem.sfu.ca/forestry/gla/), which calculates the proportions of direct and diffuse radiation beneath the canopy relative to those above the canopy. The output of GLA includes the following light variables (definitions taken from software manual, Frazer et al. 1999):

"Percentage of canopy openness is the percentage of open sky seen from beneath a forest canopy. This measure is computed from the hemispherical photograph only, and does not take into account the influence of the surrounding topography"

"Leaf area index 4Ring is the effective leaf area index integrated over the zenith angles 0 to 60°"

"Leaf area index 5Ring is the effective leaf area index integrated over the zenith angles 0 to 75°"

"Transmitted direct is the amount of direct solar radiation transmitted by the canopy in mol m^-2^ d^-1^"

"Transmitted diffuse is the amount of diffuse solar radiation transmitted by the canopy in mol m^-2^ d^-1^"

"Transmitted total is the sum of transmitted direct and transmitted diffuse"

"Percentage transmitted direct is the ratio of transmitted direct to above direct mask (defined as the amount of direct radiation incident on a horizontal or tilted surface) multiplied by 100%"

"Percentage transmitted diffuse is the ratio of transmitted diffuse to above diffuse mask (defined as the amount of diffuse radiation incident on a horizontal or tilted surface) multiplied by 100%"

"Percentage transmitted total is the ratio transmitted total to above total mask (defined as the sum of above direct mask and above diffuse mask) multiplied by 100%"

Photographs were analyzed twice so that threshold values were averaged before running the program. To document the light environment of the forest, 40 photographs were taken at random points on each habitat type at each site, these represent the control points in Suppl. material 4. Random numbers were used to select the location along the trail systems and the camera was located at the average *Geonoma macrostachys* crown height (approximately 90 cm). Control points were not taken at LLNR since the lack of a trail system made this task impractical. Suppl. material 4 presents the raw data, while Table 5 is a summary table showing mean values and standard deviations for three representative light measurements. Only the leaf area index was significantly different between local morphotypes at ACTS.

## Geographic coverage

### Description

See Fig. 1

## Taxonomic coverage

### Description

*Geonoma macrostachys* Mart. belongs to tribe Geonomateae within the Arecaceae family. It has been described as a species complex with several varieties, subspecies or morphotypes. Synonyms include: *Geonoma acaulis*, Geonoma acaulis subsp. tapajotensis, *Taenianthera oligosticha*, *Geonoma tamandua*, *Geonoma supracostata*, *Geonoma atrovirens*, *Geonoma ecuadoriensis*, and *Geonoma poiteuana* (Henderson 2011).

## Temporal coverage

### Notes

Fieldwork was conducted between January and August 2003. Soil texture and nutrient analyses in the laboratory were conducted between September and December 2003.

## Usage rights

### Use license

Creative Commons CCZero

### IP rights notes

This dataset can be freely used provided it is cited.

## Data resources

### Data package title

Edaphic and light conditions for Geonoma macrostachys

### Resource link

http://julissaroncal.wordpress.com/data-resources/

### Number of data sets

2

### Data set 1.

#### Data set name

Soil

#### Data format

.xls

#### Number of columns

27

#### Description

Soil data for three Peruvian tropical forests where *Geonoma macrostachys* occurs. Samples taken from outside the transect are labeled by the trail and meters from its starting point.

**Data set 1.** 

Column label	Column description
Location	One of the three study sites. EBCC=Cocha Cashu Biological Station, LLNR=Loma Linda Native Reserve, ACTS=Amazon Conservatory of Tropical Studies
Habitat	One of the following categories visually identified in the field: floodplain, terra firme, white soil, red soil
Plot	Transect and plot number from where soil sample was collected. C=EBCC, L=LLNR, A=ACTS
pH	pH
%sand	percentage of sand
%silt	percentage of silt
%clay	percentage of clay
Textural class	Soil textural class following the USDA textural triangle system
slope	plot inclination as measured in the field using a clinometer in the direction of the transect
Ca (lb/A)	Calcium in pounds per acre
Ca (cmol/Kg)	Calcium in cmol per kilogram
Mg (lb/A)	Magnesium in pounds per acre
Mg (cmol/Kg)	Magnesium in cmol per kilogram
P (lb/A)	Phosphorous in pounds per acre
P (cmol/Kg)	Phosphorous in cmol per kilogram
K (lb/A)	Potassium in pounds per acre
K (cmol/Kg)	Potassium in cmol per kilogram
Zn (lb/A)	Zinc in pounds per acre
Zn (cmol/Kg)	Zinc in cmol per kilogram
Mn (lb/A)	Manganese in pounds per acre
Mn (cmol/Kg)	Manganese in cmol per kilogram
Cu (lb/A)	Coper in pounds per acre
Cu (cmol/Kg)	Copper in cmol per kilogram
B (lb/A)	Boron in pounds per acre
B (cmol/Kg)	Boron in cmol per kilogram
Na (lb/A)	Sodium in pounds per acre
Na (cmol/Kg)	Sodium in cmol per kilogram

### Data set 2.

#### Data set name

Light

#### Data format

.xls

#### Number of columns

13

#### Description

Light conditions associated with the occurrence of *Geonoma macrostachys* at three Peruvian forests.

**Data set 2.** 

Column label	Column description
Location	One of the three study sites. EBCC=Cocha Cashu Biological Station, LLNR=Loma Linda Native Reserve, ACTS=Amazon Conservatory of Tropical Studies
Habitat	One of the following categories visually identified in the field: floodplain, terra firme, white soil, red soil
Plot	Transect and plot number from where soil sample was collected. C=EBCC, L=LLNR, A=ACTS
Morphotype	One of the following identified in the field: acaulis, macrostachys, small morphotype, large morphotype
% canopy openness	Percentage of open sky seen from beneath a forest canopy. This measure is computed from the hemispherical photograph only, and does not take into account the influence of the surrounding topography
Leaf area index (4Ring)	The effective leaf area index integrated over the zenith angles 0 to 60°
Leaf area index (5Ring)	The effective leaf area index integrated over the zenith angles 0 to 75°
Transmitted Direct	The amount of direct solar radiation transmitted by the canopy in mol m^-2^ d^-1^
Transmitted Diffuse	The amount of diffuse solar radiation transmitted by the canopy in mol m^-2^ d^-1^
Transmitted Total	The sum of transmitted direct and transmitted diffuse
% Transmitted Direct	The ratio of transmitted direct to above direct mask (defined as the amount of direct radiation incident on a horizontal or tilted surface) multiplied by 100%
% Transmitted Diffuse	The ratio of transmitted diffuse to above diffuse mask (defined as the amount of diffuse radiation incident on a horizontal or tilted surface) multiplied by 100%
% Transmitted Total	The ratio transmitted total to above total mask (defined as the sum of above direct mask and above diffuse mask) multiplied by 100%

## Supplementary Material

Supplementary material 1 Occurence data for Geonoma macrostachys Mart. morphotypes on transects at three Peruvian forests Data type: occurrences
Brief description: Raw data of morphotype numbers along each of the 38 transects established in Peru.
File: oo_5751.xls Julissa Roncal, Christine Bacon, Ines Angulo, Celso Narino

Supplementary material 2 Trail system at The Amazon Conservatory of Tropical Studies, Loreto, Peru Data type: trail map
Brief description: As of March 2003.
File: oo_5778.jpg Julissa Roncal and Ines Angulo

Supplementary material 3 Soil data for three Peruvian tropical forests where Geonoma macrostachys occurs Data type: ecological
Brief description: Raw soil data. Samples taken from outside the transect are labeled by the trail followed by the meters from its starting point. Locality acronyms as in Table 1.
File: oo_5793.xls Julissa Roncal

Supplementary material 4 Light conditions associated with the occurrence of Geonoma macrostachys at three Peruvian forests Data type: ecological
Brief description: Locality acronyms as in Table 1.
File: oo_5795.xls Julissa Roncal

## Figures and Tables

**Table 1.** Geographic location of study sites.

Study sites	Peruvian department	Latitude and Longitude	Altitude (m.a.s.l.)	Mean annual temperature (°C)	Total annual precipitation (mm)	Reference
Amazon Conservatory of Tropical Studies (ACTS)	Loreto	03°15’S 72°54’W	130	25.9	2,948	Vasquez 1997
Loma Linda Native Reserve (LLNR)	Pasco	10°19’S 75°03’W	350	23.2	7,106	Anonymous 1990
Cocha Cashu Biological Station (EBCC)	Madre de Dios	11°50’S 71°23'W	400	24.1	2,080	Terborgh 1990

**Table 2.** Transect location where edaphic and light conditions were measured. GPS coordinates and trail system (trail number: meters from its origin) indicate the start of each transect. No trail system was available at LLNR. Locality acronyms as in Table 1.

Transect	GPS coordinates	Trail system	Direction
**EBCC**			
CT1	11°53.37S, 71°24.39W	trail7:1632	N
CT2	11°53.02S, 71°24.45W	trail10:00	79°
CT3	11°53.13S, 71°23.92W	trail35:00	20°
CT4	11°52.26S, 71°24.85W	trail59:1800	84°
CT6	11°50.46S, 71°23.26W	trail27:intersection with "playa bonita"	S
CT7	11°54.01S, 71°24.05W	crossing river:200	N
CT8	11°54.21S, 71°24.14W	crossing river:700	N
CT9	11°54.53S, 71°24.11W	crossing river:1300	E
CT16	11°54.44S, 71°24.09W	crossing river:1100	E
CT17	11°52.65S, 71°24.07W	trail11:300	N
CT18	11°53.71S, 71°24.69W	trail27:1550	53°
**LLNR**			
LT1	10°19.03S, 75°04.77W		W
LT2	10°19.43S, 75°05.20W		310°
LT3	10°19.33S, 75°05.17W		310°
LT4	10°19.42S, 75°04.60W		290°
LT5	10°19.49S, 75°04.47W		140°
LT6	10°19.70S, 75°04.15W		20°
LT7	10°19.72S, 75°03.87W		150°
LT8	10°19.45S, 75°05.38W		160°
LT9	10°18.97S, 75°04.98W		250°
LT10	10°18.92S, 75°04.88W		140°
LT11	10°18.62S, 75°04.95W		330°
LT12	10°18.77S, 75°04.93W		110°
**ACTS**			
AT1	03°15.34S, 72°55.00W	CQT:200	23°
AT2	03°15.27S, 72°54.83W	QT:925	158°
AT3	03°15.24S, 72°54.78W	QT:1100	71°
AT4	03°15.11S, 72°54.70W	QT:1400	71°
AT5	03°14.78S, 72°54.61W	TT:250	S
AT6	03°15.02S, 72°54.71W	DT:175 a 200m	210°
AT7	03°14.94S, 72°54.72W	DT:275 a 20m	S
AT8	03°14.87S, 72°54.55W	QT:2075	340°
AT9	03°14.86S, 72°54.40W	MT:200	E
AT10	03°15.26S, 72°54.47W	NT:1150	E
AT11	03°15.40S, 72°54.16W	CWT:1300	W
AT12	03°14.96S, 72°53.96W	TAMBOS:700	W
AT13	03°15.43S, 72°54.73W	D:275	W
AT14	03°14.75S, 72°54.54W	LNT:700	S

**Table 3.** Mean values and standard deviation (in parenthesis) for 13 edaphic variables describing the two main habitats found at each study site. Locality acronyms as in Table 1. FP=floodplain, TF=terra firme, WS=white soil, RS=red soil, n=number of 10×10 m plots sampled.

edaphic variable	ACTS	LLNR	EBCC
	FP (n=45) / TF (n=33)	WS (n=30) / RS (n=46)	FP (n=59) / TF (n=28)
pH	4.22 (±0.26) / 4.21 (±0.29)	4.27 (±0.28) / 4.22 (±0.22)	6.65 (±0.5) / 4.60 (±0.62)
% sand	27.65 (±12.7) / 45.53 (±7.17)	79.47 (±10.29) / 49.1 (±11.4)	31.83 (±17.14) / 71.63 (±11.48)
% clay	47.52 (±12.41) / 37.27 (±8.65)	8.37 (±7.07) / 29.15 (±9.16)	39.41 (±13.38) / 12.89 (±7.01)
Inclination	2.07 (±2.57) / 5.61 (±4.43)	3.8 (±5.4) / 21.87 (±9.76)	1.06 (±1.13) / 7.38 (±7.69)
Ca (cmol/kg)	0.32 (±0.25) / 0.27 (±0.38)	0.1 (±0.03) / 0.26 (±0.41)	6.42 (±1.42) / 0.51 (±0.88)
Mg (cmol/kg)	0.176 (±0.094) / 0.111 (±0.08)	0.049 (±0.019) / 0.155 (±0.151)	1.297 (±0.405) / 0.163 (±0.208)
P (cmol/kg)	0.003 (±0.004) / 0.002 (±0.002)	0.005 (±0.004) / 0.007 (±0.005)	0.09 (±0.057) / 0.014 (±0.006)
K (cmol/kg)	0.097 (±0.025) / 0.069 (±0.02)	0.059 (±0.023) / 0.144 (±0.026)	0.169 (±0.037) / 0.085 (±0.034)
Zn (cmol/kg)	0.007 (±0.002) / 0.006 (±0.002)	0.008 (±0.003) / 0.011 (±0.003)	0.006 (±0.003) / 0.008 (±0.004)
Mn (cmol/kg)	0.08 (±0.086) / 0.057 (±0.067)	0.001 (±0.002) / 0.026 (±0.037)	0.115 (±0.034) / 0.186 (±0.198)
Cu (cmol/kg)	9.29×10^-4^ (±5.16×10^-4^) / 1.93×10^-4^ (±3.44×10^-4^)	4.19×10^-5^ (±1.66×10^-4^) / 7.47×10^-4^ (±4.46×10^-4^)	7.73×10^-4^ (±3.83×10^-4^) / 4.83×10^-4^ (±3.98×10^-4^)
B (cmol/kg)	0.007 (±0.003) / 0.009 (±0.004)	0.013 (±0.002) / 0.013 (±0.002)	0.01 (±0.009) / 0.014 (±0.011)
Na (cmol/kg)	0.067 (±0.011) / 0.06 (±0.009)	0.058 (±0.011) / 0.082 (±0.021)	0.064 (±0.021) / 0.041 (±0.012)

**Table 4.** Mean values, standard deviations, and T-test statistics between local morphotypes for only significantly different edaphic variables, arranged by study site. * P<0.05, ** P<0.01, *** P<0.001.

	acaulis or small morphotype	macrostachys or large morphotype	T-test
	mean±S.D.	mean±S.D.	
**ACTS**			
% sand (n=39,31)	25.173±8.996	43.911±10.92	-7.873***
% clay (n=40,40)	50.613±9.543	35.5±9.040	7.271***
Inclination (n=28,38)	2.57±3.49	5.26±4.22	-2.75**
Mg (cmol/kg, n=40,40)	0.1755±0.0908	0.119±0.0869	2.845**
K (cmol/kg, n=40,40)	0.0986±0.0256	0.0709±0.02	5.403***
Cu (cmol/kg, n=28,38)	9.2×10^-4^±4.45×10^-4^	2.55×10^-4^±4.27×10^-4^	6.141***
B (cmol/kg, n=40,40)	6.91×10^-3^±3.3×10^-3^	8.76×10^-3^±3.62×10^-3^	-2.386*
Na (cmol/kg, n=28,38)	6.81×10^-2^±1.08×10^-2^	6.05×10^-2^±0.99×10^-2^	2.959**
**LLNR**			
% sand (n=40,40)	73.069±14.942	49.681±12.836	7.509***
% clay (n=40,40)	12.931±10.574	28.675±10.071	-6.819***
Inclination (n=40,40)	7.80±10.17	21.55±9.54	-6.235***
Mg (cmol/kg, n=35,40)	5.08×10^-2^±1.98×10^-2^	0.1572±0.1505	-2.461*
P (cmol/kg, n=35,40)	3.99×10^-3^±3.96×10^-3^	7.41×10^-3^±4.55×10^-3^	-2.389*
K (cmol/kg, n=35,40)	6.3×10^-2^±2.68×10^-2^	0.144±2.58×10^-2^	-5.774***
Zn (cmol/kg, n=35,40)	8.2×10^-3^±2.98×10^-3^	1.06×10^-2^±3.22×10^-3^	-3.766***
Cu (cmol/kg, n=40,40)	1.82×10^-4^±3.08×10^-4^	7.32×10^-4^±5.02×10^-4^	-5.906***
Na (cmol/kg, n=40,40)	6.46×10^-2^±1.71×10^-2^	8.09×10^-2^±2.12×10^-2^	-3.795***
**EBCC**			
pH (n=44,43)	6.65±0.50	5.46±1.12	6.883***
% sand (n=44,43)	33.183±17.727	52.088±25.254	-4.272***
% clay (n=44,43)	38.697±14.214	25.743±16.726	4.099***
Inclination (n=44,43)	1.13±1.22	4.54±6.57	-3.601***
Ca (cmol/kg, n=44,43)	6.329±1.302	3.252±3.036	7.405***
Mg (cmol/kg, n=44,43)	1.3±0.3989	0.702±0.6345	6.45***
P (cmol/kg, n=44,43)	9.24×10^-2^±6.03×10^-2^	3.95×10^-2^±3.84×10^-2^	5.562***
K (cmol/kg, n=44,43)	0.1658±3.47×10^-2^	0.1281±5.78×10^-2^	4.405***
Mn (cmol/kg, n=44,43)	0.1136±3.6×10^-2^	0.1539±0.145	-2.152*
B (cmol/kg, n=44,43)	7.68×10^-3^±7.39×10^-3^	1.45×10^-2^±1.05×10^-2^	-3.39***
Na (cmol/kg, n=38,38)	6.15×10^-2^±1.92×10^-2^	5.03×10^-2^±1.71×10^-2^	2.679**

**Table 5.** Mean values, standard deviation, and test statistics for *Geonoma macrostachys* morphotypes and habitats for three light variables measured using hemispherical photography. F values given for ACTS and EBCC are from one-way ANOVA tests, and T values for LLNR are from independent samples t-tests. Morphotypes and habitats sharing the same letter are not significantly different at the 0.05 level after Bonferroni pairwise comparisons of means. n=number of hemispherical photos, ns=non significant, *P<0.05.

	acaulis or small morphotype	macrostachys or large morphotype	floodplain	terra firme	F or T
	mean±S.D.	mean±S.D.	mean±S.D.	mean±S.D.	
**ACTS**	n=40	n=40	n=40	n=40	
% canopy openness	7.119±1.236	6.545±1.147	6.664±1.21	7.09±1.003	2.584ns
leaf area index 5ring	3.032±0.359 (a)	3.235±0.331 (b)	3.147±0.327 (a,b)	3.028±0.268 (a)	3.804*
total transmitted light (mol m^-2^ d^-1^)	6.27±1.283	5.735±1.234	5.917±1.591	6.048±1.061	1.19ns
**LLNR**	n=40	n=40			
% canopy openness	7.603±1.28	7.632±1.257	_	_	0.103ns
leaf area index 5ring	2.912±0.313	2.807±0.283	_	_	1.576ns
total transmitted light (mol m^-2^ d^-1^)	6.148±1.429	5.993±1.154	_	_	0.533ns
**EBCC**	n=44	n=39	n=40	n=40	
% canopy openness	6.622±1.15	6.806±1.237	6.695±1.689	7.175±1.173	1.414ns
leaf area index 5ring	3.093±0.342	2.98±0.242	3.069±0.376	2.928±0.285	2.453ns
total transmitted light (mol m^-2^ d^-1^)	5.744±1.185	5.803±1.323	5.678±1.461	5.876±1.176	0.173ns
