# Supplementary figures and images for: Edaphic and light conditions of sympatric plant morphotypes in western Amazonia
Source: Biodivers Data J. 2014 May 10;(2):e1078. doi: 10.3897/BDJ.2.e1078 (PMC4040399; doi:10.3897/BDJ.2.e1078)

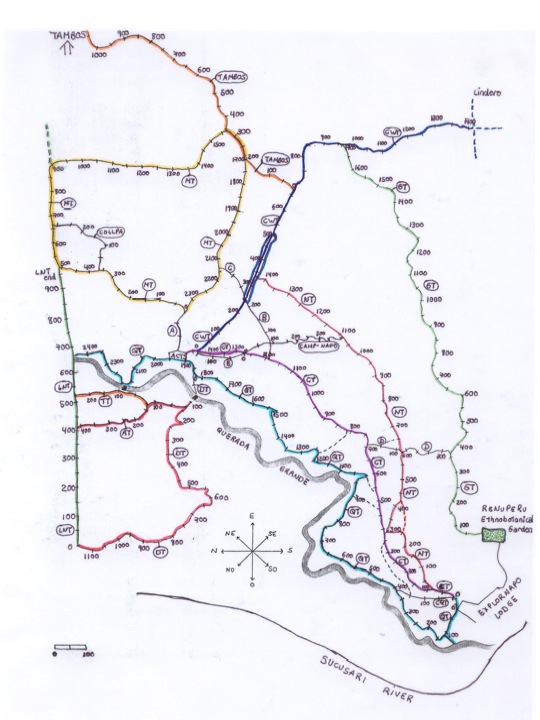

Supplement: Supplementary material 2 — Trail system at The Amazon Conservatory of Tropical Studies, Loreto, Peru [file biodiversity_data_journal-2-e1078-s002.jpg]
